# Comparison of surgical conditions in 2 different anesthesia techniques of esmolol-induced controlled hypotension in breast reduction surgery

**DOI:** 10.1097/MD.0000000000006254

**Published:** 2017-03-10

**Authors:** Ahmet Besir, Bahanur Cekic, Dilek Kutanis, Ali Akdogan, Murat Livaoglu

**Affiliations:** a Department of Anesthesiology and Critical Care, Faculty of Medicine; b Department of Plastic and Reconstructive Surgery, Karadeniz Technical University, Trabzon, Turkey.

**Keywords:** anesthesia technique, breast reduction surgery, controlled hypotension

## Abstract

**Background:**

Breast reduction surgery is a common cosmetic surgery with a high incidence of blood loss and transfusion. In this surgery, the reduction of blood loss related to surgical manipulation and the volume of resected tissue is a target. In the present study, we compared the effects of esmolol-induced controlled hypotension on surgical visibility, surgical bleeding, and the duration of surgery in patients anesthetized with propofol/remifentanil (PR) or sevoflurane/remifentanil (SR).

**Methods:**

Patients in the American Society of Anesthesiologists I/II risk group undergoing breast reduction surgery were prospectively randomized into PR (n = 25) and SR (n = 25) groups. Controlled hypotension was induced with esmolol in both groups. During the intraoperative period, the heart rate (HR), mean arterial pressure (MAP), operation duration, volume of intraoperative blood loss, volume of blood received through postoperative drains, volume of resected tissues, and surgical area bleeding score were recorded.

**Results:**

The duration of operation in the incisional period was shorter in group PR compared to group SR (*P* = 0.04). The change in HR was lower in incision and hemostasis periods in the group PR compared to the group SR (*P* < 0.001). Total intraoperative intraoperative bleeding volume and volume of blood received through drains on postoperative postoperative day 1, day 2, and in total were found to be significantly lower in group PR compared to group SR. Surgical visibility scoring was more effective in group PR compared to SR.

**Conclusion:**

In the breast reduction surgery performed under esmolol-induced controlled hypotension, the effect of propofol + remifentanil anesthesia on the duration of incisional surgery, surgical visibility, and volume of surgical blood loss was more reliable and effective compared to that of sevoflurane + remifentanil, which seems to be an advantage.

## 1 Introduction

Breast reduction surgery is performed frequently due to various social challenges, as well as functional complaints such as back, shoulder and neck ache, hygiene problems, and difficulty with movement. After surgery, areola problems such as skin and fat necrosis, hypertrophic scarring, sensory loss, deformation, retraction, and malposition, as well as severe complications such as infection and hematoma requiring reoperation can be observed.^[1]^ The incidence rate of such complications varies between 10% and 30%.^[2]^

Bleeding at the surgical site contributes to an increase in these complications. The primary aims in surgery are to enable surgical visibility with minimal bleeding and to minimize the duration of surgery. In recent years, a controlled hypotensive anesthesia method has often been preferred in various surgical procedures where excessive bleeding is expected and visibility is limited in order to enhance surgical visibility and reduce microtrauma related to reduction in surgical manipulation, as well as surgical bleeding associated with the volume of resected tissue.^[3,4]^ The beneficial effects of controlled hypotension on the volume of intraoperative blood loss have been described in general surgery, neurosurgery, and orthopedic surgery.^[4]^ There are a limited number of studies in the literature regarding the use of the controlled hypotension method in breast reduction operations where excessive bleeding is expected (mean 700 to 800 mL).^[1,5,6]^

For controlled hypotension, inhalational or intravenous anesthetics are used, with esmolol, a short-acting β1 adrenoreceptor antagonist, being an adjuvant drug of choice in recent years. Esmolol has been suggested to be more reliable and effective because it does not lead to reflex tachycardia and causes less intraoperative hemorrhaging.^[4,7,8]^ In the literature, propofol anesthesia has been shown to be more effective than isoflurane or sevoflurane anesthesia in terms of its effects on surgical bleeding, the operation site, and the duration of operation.^[9–12]^

The primary aim of our this study was to compare the effects of 2 diverse anesthesia methods, namely propofol/remifentanil (PR) and sevoflurane/remifentanil (SR) anesthesia of esmolol-induced controlled hypotension on the surgical visibility, surgical bleeding, and duration of operation in breast reduction surgery.

## 2 Materials and methods

The study was started after obtaining Local Ethics Committee approval No. 2012/187 from Karadeniz Technical University Faculty of Medicine and the written consent of patients. The study was performed on a total of 50 patients in the American Society of Anesthesiologists I–II risk group, who had various social, functional, and cosmetic complaints were aged between 18 and 65 and planned to undergo a breast reduction operation. The study power was determined at 85.6% to 90%. The study was conducted between 2 groups who were randomized into PR (n = 25) and SR (n = 25) groups using the sealed envelope method and then underwent breast reduction operation in the plastic reconstructive surgery room. The intraoperative surgery period was divided into 3 periods; incision (I), hemostasis (H), and closure (C). Incision period was used for deepithelialization of the skin, the hemostasis period for operative area hemostasis, and the closure period for wound closure and creation of the areolar complex.

Patients with anticoagulant drug use, bleeding diathesis history, uncontrolled systemic diseases (eg, diabetes mellitus, asthma, and chronic obstructive pulmonary disease), resting blood pressure values of systolic >140 mm Hg or diastolic >90 mm Hg, coronary insufficiency (myocardial infarction), neurological deficits, peripheral vascular disease, renal and liver disease history, morbid obesity, chronic drug use, or drug allergy history were excluded. The data obtained were recorded by an anesthesiologist outside the team and interpreted after surgery by the team.

For the routine monitoring of patients in the surgery room electrocardiography, heart rate (HR), noninvasive blood pressure, peripheral oxygen saturation (SpO_2_), end-tidal CO_2_ (EtCO_2_) (Spacelabs Medical, WA), bispectral index (BIS; Aspect Medical Systems, Inc. Newton, MA), and neuromuscular monitoring (train-of-four [TOF] method; TOF-Watch SX, Dublin, Ireland) were assessed. The HR, mean arterial pressure (MAP), SpO_2_, BIS score, and EtCO_2_ values of patients were recorded every 5 minutes before and after the surgical incision, at the end of the operation and after extubation; the total duration of anesthesia and duration of operation including incision, hemostasis, closure, and total values were also recorded.

A vascular line was prepared for anesthesia induction and saline crystalloid solution replacement. Within 30 minutes before the surgery, 5 mL/kg/h 0.9% NaCl was administered followed by premedication with 0.03 mg/kg midazolam IM (1 hour prior to surgery). Following a 0.5 mg/kg loading dose of esmolol IV to both groups 1 minute prior to induction, a 0.1 to 0.3 mg/kg/min esmolol infusion was administered so that the MAP would be between 50 and 65 mm Hg after induction. Hypotensive anesthesia was administered as a standard in both groups during the incision period, and the esmolol infusion was stopped during the hemostasis and scar closure stages. After 3 minutes of preoxygenation, 1 mg/kg lidocaine was administered 60 seconds before propofol injection to prevent pain due to injection. Anesthesia induction was performed with 2 to 3 mg/kg propofol, 1 μg/kg fentanyl, and 0.6 mg/kg rocuronium IV for muscle relaxation, and once the BIS score was reduced to below 60 and the TOF response disappeared, endotracheal intubation was performed. Patients were ventilated using mechanical ventilation at 6 mL/kg tidal volume, 10 to 12 breaths/min, and 32 to 35 mm Hg EtCO_2_. After induction, both groups received a total gas flow of 4 L/min, a 1:1 O_2_/air rate, and continuous 0.2 μg/kg/min remifentanil for the maintenance of anesthesia, keeping the BIS between 40 and 60. Group SR received 1% to 3% sevoflurane, whereas group PR received 100 to 200 μg/kg/min propofol infusion maintenance of anesthesia. In the case when the MAP and HR were 20% above the baseline despite having reached the higher value of the esmolol infusion rate (0.3 mg/kg/min), 10 to 20 mg esmolol were administered every 5 to 10 minutes over the course of 1 minute. When the BIS was >60 without hypertension or tachycardia, 0.5 to 1 mg/kg bolus propofol was administered in group PR, and sevoflurane concentration was titrated in 1% increments up to a maximum of 3%. An HR below 45 beats/min was considered as bradycardia, and 0.5 mg atropin IV were administered at repeated doses (maximum 3 mg) if not sufficient then the esmolol infusion was reduced in doses of 0.1 mg/kg/min. A MAP below 50 mm Hg was considered hypotension; in that case, 1st, the dose of the hypotensive agent was reduced if there was no improvement after 5 minutes, then a bolus of 10 mg ephedrine was administered IV. This bolus was repeated twice if the MAP did not remain >50 mm Hg for 5 minutes. If this goal was not reached, the hypotension was considered as refractory, and we administered positive inotropic agents. These cases were excluded from the study. All the possible complications were recorded.

When the TOF was 25% at the end of the operation, neuromuscular block was recovered with 0.05 mg/kg neostigmin and 0.02 mg/kg atropine, and patients with sufficient spontaneous respiration (TOF above 75% and a BIS score >80) were extubated. After extubation, all patients were transferred to the postanesthetic care unit and observed for possible early complications for an hour. At the end of this observation, patients with a modified Aldrete score of over 9 were sent to their rooms.^[13]^

The total volume of intraoperative bleeding was measured by using a suction canister and weighing gauzes. The weights of the resected right and left breast tissues were recorded individually. The intraoperative surgical bleeding site evaluation was examined with a category scale adapted by Fromme et al (Table 1).^[14]^ The total amounts of esmolol and remifentanil consumed were recorded. The hemoglobin (Hb) and hematocrit levels of patients were recorded at the preoperative and postoperative 24th hour, and the amount of blood received through the drains attached to both breasts was recorded at the postoperative 48th hour. The anesthesiologist evaluating all the collected data was blinded to the anesthetic technique.

**Table 1:**
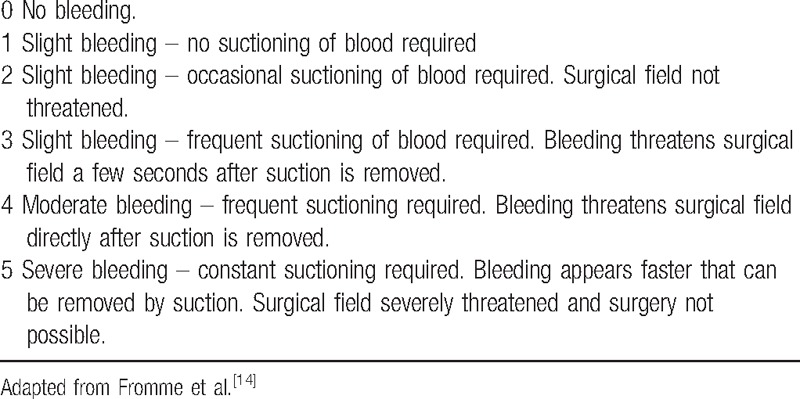
Intraoperative surgical area scale.

Although evaluating the findings obtained from the study, the Statistical Package Program was used for statistical analyses. The Kolmogorov–Smirnov distribution test was used for examination of the normal distribution addition the descriptive statistical methods (frequency, percentage, mean, and standard deviation).

The Pearson chi square test and Fisher exact test were used for the comparison of nominal data. For the comparison of digital data, the Mann–Whitney *U* test was used to compare parameters between the 2 groups. The Wilcoxon signed rank test was used in the intragroup comparison of parameters. Pearson correlation analysis was used for the examination of the relationship between the volume of resected breast tissue, duration of operations, and laboratory findings. The power of 50 cases, 25 in each group, was calculated as 91%.

Results were evaluated within the 95% reliability index with *P* < 0.05 being significant.

## 3 Results

The study was conducted on a total of 50 patients divided into group PR (n = 25) and group SR (n = 25), both of which were to have breast reduction surgery. No cases were excluded from the study. The age, American Society of Anesthesiologists classification, body mass index, total operation, and anesthesia durations of the groups were similar whereas the operation duration during the intraoperative incision time was significantly shorter in group PR (*P* = 0.04; Table 2)

**Table 2:**
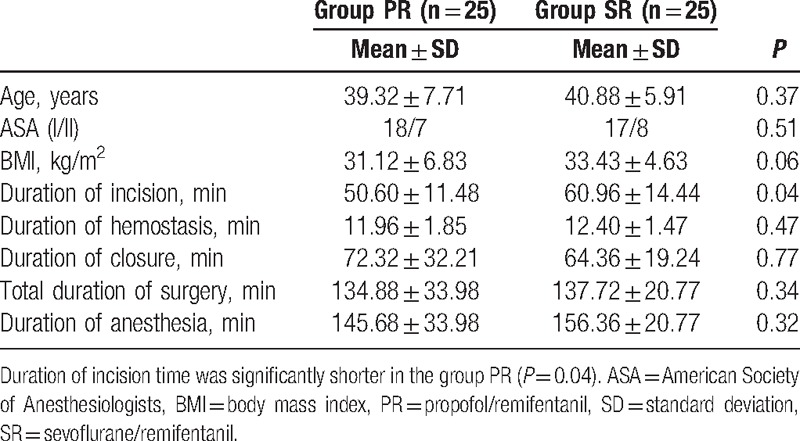
Demographic characteristics and durations of surgery and anesthesia.

**Figure 1:**
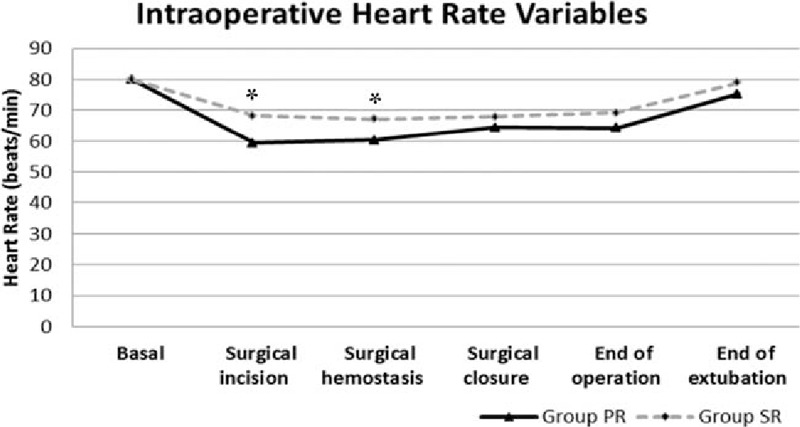
Intraoperative heart rate variables.

**Figure 2:**
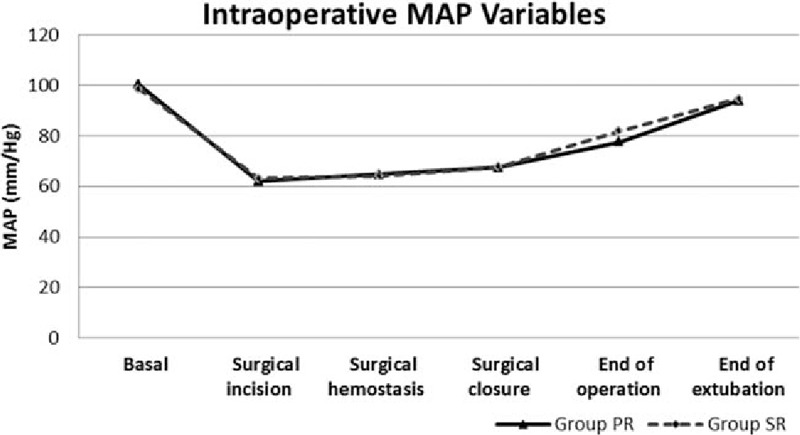
Intraoperative mean arterial pressure (MAP) variables.

The intraoperative MAP changes of patients were similar in terms of periods, but the change in HR was significantly lower in group PR during the incision and hemostasis periods compared to group SR (Figs. 1 and 2).

Intraoperative remifentanil and esmolol consumption and the total volume of resected breast tissue were also similar in group PR and the group SR (Table 3). The groups appeared to be similar in terms of SpO_2_ and BIS levels (*P* > 0.05).

**Table 3:**
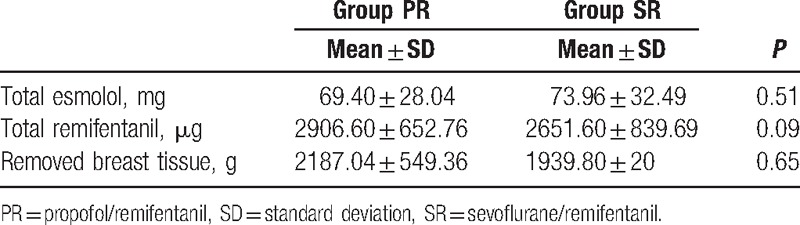
Intraoperative drug consumption and resected breast tissue.

In the comparison of the 2 groups, although it was higher in group PR than in group SR, there was no significant difference in the change between preoperative and postoperative Hb (11.02 ± 0.85, 10.92 ± 1.00, *P* = 0.48, respectively) or hematocrit (33.98 ± 2.59, 32.37 ± 2.94, *P* = 0.29, respectively). Postoperative Hb levels were lower than preoperative Hb levels in both groups (*P* = 0.0001).

The total intraoperative bleeding volume, volume of blood received through the drains on days 1 and 2, and total volume of blood received through the drains were significantly lower in group PR (Table 4).

**Table 4:**
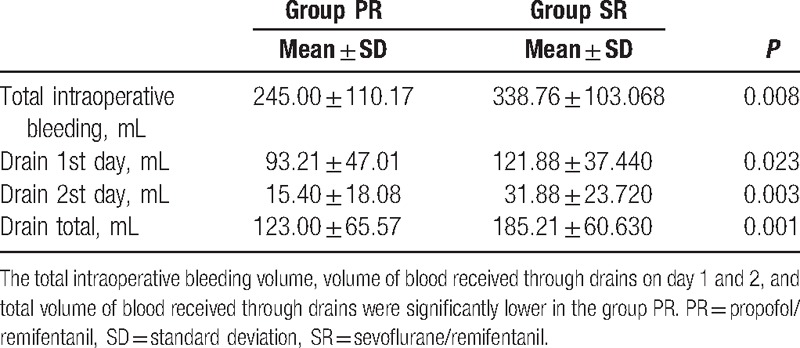
Intraoperative and postoperative bleeding data.

The intraoperative surgical bleeding site scale, the anesthesia technique that was evaluated by a blinded surgeon, is shown on Table 5. No complications were seen in the postoperative period.

**Table 5:**
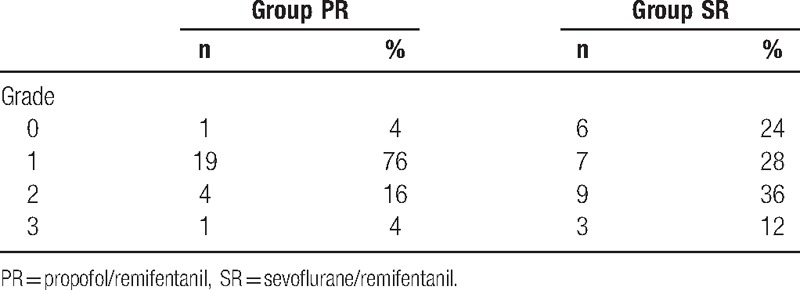
Surgical area scale.

## 4 Discussion

In our study, we found that the incisional surgical duration was shorter, the quality of surgical visibility was increased, and the amount of intraoperative and postoperative surgical blood loss was reliably and effectively decreased in the propofol + remifentanil anesthesia group compared to the sevofluran + remifentanil group in breast reduction operation with esmolol-controlled hypotension.

In breast reduction operations where excessive bleeding is expected, complications such as bleeding and hematoma can be seen in the postoperative period. The aim is to enable surgical visibility with minimal bleeding and thus decrease the complications. Surgical bleeding is affected by regional capillary circulation, as well as arterial and venous pressure.^[8]^ Therefore, the controlled hypotensive anesthesia method is preferred in order to decrease bleeding and improve surgical visibility in breast reduction surgeries where a large amount of resected tissue and excessive bleeding are expected.^[3,4]^ There are many methods and techniques in the literature used for controlled hypotensive anesthesia.^[4]^ Boezaart et al^[15]^ observed that esmolol provided optimal surgical conditions when controlled hypotension was performed with esmolol and sodium nitroprusside under inhalation anesthesia in functional endoscopic sinus surgery (ESS). In the study in which Srivastava et al^[8]^ compared esmolol with nitroglycerine, esmolol was found to enable optimal surgical conditions, reduce intraoperative bleeding, and cause less tachycardia throughout the operation. In our study, we also chose to use esmolol, a β1 adrenoceptor antagonist, in order to provide controlled hypotension, due to its rapid release, short half-life, and not causing tachycardia or rebound hypertension, as well as its easy titration capability.

The use of inhalation anesthetics alone is not recommended for controlled hypotension because of their respective adverse effects. Therefore, inhalation agents have to be used with adjuvant agents.^[4]^ Superiority between hypotensive anesthesia with inhalation agents is questionable in the literature.^[16–18]^ Therefore in our study we preferred sevoflurane, which is frequently used in our clinic.

Anesthetics agents can affect the amount of blood loss through various pharmacological effects on the degree of vasodilatation and HR.^[9]^ Both propofol and inhalation agents have a dose-dependent vasodilatory effect.^[19,20]^ When compared to isoflurane, an inhalation agent, sevoflurane generally does not change the HR whereas propofol inhibits the baroreflex and thus leads to bradycardia, and propofol suppresses more cardiac output than sevoflurane.^[19–21]^In a study by Ahn et al,^[9]^ HR was significantly lower in the PR group compared to the SR group, whereas there was no difference between MAP values in patients who had no cardiovascular disease but received controlled hypotension during ESS. The researchers also observed a lower amount of intraoperative blood loss in the PR group, which had lower intraoperative HR. Nair et al^[22]^ evaluated the effect of beta-blocker premedication on the surgical field during ESS. They found that surgical bleeding scores were correlated with HR, but not with MAP. A low HR leads to the overfilling of veins and decreased venous leakage at the surgical site. In addition to bradycardia resulting from propofol-inhibited baroreflex, it is thought that an opioid such as remifentanil, which is used in infusion constantly, might also increase such an effect. In our study, the consumption of infused remifentanil was the same in both groups, whereas HR was significantly lower in the PR group, including the I and H periods compared to the SR group. MAP changes were not statistically significant in either groups.

A limited number of studies in the literature have reported the use of controlled hypotension in breast reduction surgeries where the risk of blood loss and the possibility of transfusion are high.^[1,3,5]^

In a study evaluating hemodynamic changes and surgical ischemia with a sodium nitroprusside infusion in hypertrophic breast removal surgery, Vazeery et al^[5]^ observed that the hypotensive group did not need a transfusion. Kop et al^[1]^ compared normotension with controlled hypotension in breast reduction surgery. They found a positive correlation between blood pressure and the amount of blood loss. They reported that blood loss was reduced by more than 50% with controlled hypotension in the incisional surgery period. This reduction was achieved with a reduction in systolic blood pressure of 20% to 25%. In addition, the amount of blood received through drains during the postoperative period was found to be significantly lower in the hypotensive group in the same study. In our study, there was a 35% to 40% reduction in MAP in the surgical incision period in both groups. We also saw a significant decrease in the amount of both the intraoperative blood loss and the blood received through drains postoperative in the PR group as compared to the SR group, under hypotensive anesthesia enabled with esmolol. We believe that the reason for the reduction in intraoperative and postoperative bleeding during surgical incision in the PR group was due to a significant decrease in the HR.

Scar-site hematoma incidence following breast reduction is 0.3% to 2.6%.^[23,24]^ In the literature, the overall breast reduction complication rate has been reported to be higher, especially in those with resected breast tissue of more than 1000 g.^[6,25]^ Among these, in the study in which Hussien et al^[6]^ examined the effects of intraoperative hypotension on the development of scar-site hematoma following breast reduction, the total amount of resected breast tissue was higher than 1000 g (1215.4 vs 1104.0 g), despite not being statistically significant, in the group with hematoma development during the postoperative period (6). In our study, although the total amounts of resected breast tissue were much higher (2187.04 vs 1939.8 g), no hematoma was observed in the postoperative period. In the same study mentioned above, hypotension in the intraoperative hemostasis period was associated with the development of scar hematoma in postoperative period. In our study, hypotensive anesthesia was as not performed during the intraoperative hemostasis period nor the following closure period and no complication related to bleeding such as scar hematoma was observed in the postoperative period. The more breast tissue removed correlated more intraoperative and postoperative bleeding. We believe that hypotension during intraoperative hemostasis may increase the risk of hematoma development due to changes in blood pressure in the postoperative period.

Breast reduction surgery requires careful evaluation in terms of operation duration for the anesthesiologist, the surgeon, and the patient, as well as the possibility of blood transfusion. In this surgery, intraoperative blood loss and the possibility of transfusion can be reduced and a comfortable surgical site can be provided thanks to the controlled hypotensive anesthesia method. The early discharge of patients could in turn contribute to a reduction in hospital costs.

One of our study's limitations was the failure to calculate the rate of reaching hypotension and the percent decrease in blood pressure. Also, continuous invasive hemodynamic monitoring is recommended in hypotensive anesthesia to monitor beat to beat variation in blood pressure. Limitation of our study is measured intermittently of MAP. Another limitation, because there was no device in our clinic, was that target-controlled infusion was not used to deliver the anesthetic agents. We believe advanced studies are required to examine the effects of controlled hypotension on breast reduction surgery.

In conclusion, the PR anesthesia technique of esmolol-induced controlled hypotension reduces the duration of incisional surgery, improves surgical visibility, and decreases the amount of intraoperative and postoperative blood loss reliably and effectively compared to the SR anesthesia technique.

Therefore, we recommended the PR anesthesia technique of esmolol-induced controlled hypotension due to the reduced duration of incisional surgery, improvised surgical visibility, and effectively lost blood.
